# *Entamoeba* Encystation: New Targets to Prevent the Transmission of Amebiasis

**DOI:** 10.1371/journal.ppat.1005845

**Published:** 2016-10-20

**Authors:** Fumika Mi-ichi, Hiroki Yoshida, Shinjiro Hamano

**Affiliations:** 1 Division of Molecular and Cellular Immunoscience, Department of Biomolecular Sciences, Faculty of Medicine, Saga University, Saga, Japan; 2 Department of Parasitology, Institute of Tropical Medicine (NEKKEN), Nagasaki University, Nagasaki, Japan; Boston College, UNITED STATES

## Abstract

Amebiasis is caused by *Entamoeba histolytica* infection and can produce a broad range of clinical signs, from asymptomatic cases to patients with obvious symptoms. The current epidemiological and clinical statuses of amebiasis make it a serious public health problem worldwide. The *Entamoeba* life cycle consists of the trophozoite, the causative agent for amebiasis, and the cyst, the form responsible for transmission. These two stages are connected by “encystation” and “excystation.” Hence, developing novel strategies to control encystation and excystation will potentially lead to new measures to block the transmission of amebiasis by interrupting the life cycle of the causative agent. Here, we highlight studies investigating encystation using inhibitory chemicals and categorize them based on the molecules inhibited. We also present a perspective on new strategies to prevent the transmission of amebiasis.

## *Entamoeba histolytica* Infection and Amebiasis: Behavior of the Causative Agent and the Parasite Life Cycle

Amebiasis is caused by *Entamoeba histolytica* infection, a protozoan parasite belonging to the phylum Amoebozoa. Infected individuals show a wide range of clinical signs and can be asymptomatic or have obvious symptoms, such as diarrhea, dysentery, fever, and abdominal pains owing to invasive infection. As a consequence of the invasive infection, various extra-intestinal manifestations may also arise: for example, amebic liver, lung, or brain abscesses. Worldwide, 35–50 million symptomatic cases occur annually, leading to approximately 55,000 deaths [1]. However, only a few drugs are available, and no effective vaccines exist [2,3]. Furthermore, because of the high occurrence of asymptomatic infections, amebiasis morbidity is thought to be much higher than estimates that are only based on the number of reported symptomatic cases. These clinical and epidemiological statuses make amebiasis a serious public health problem worldwide [4].

**Fig 1:**
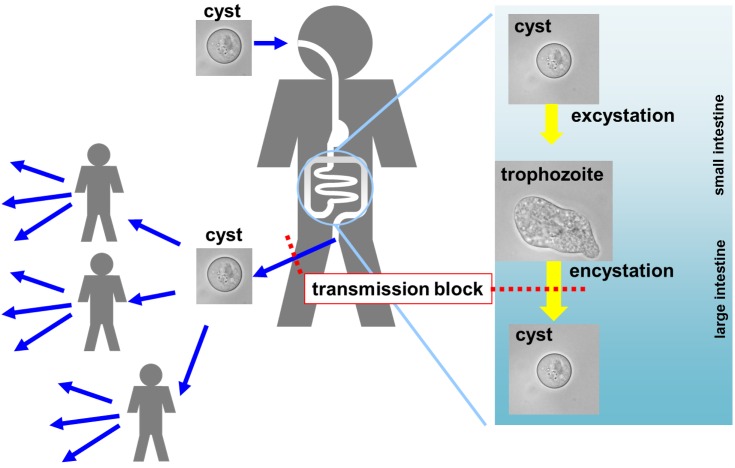
*E*. *histolytica* infection and spread of amebiasis; behavior of the causative agent and the parasite life cycle. Schematic of *E*. *histolytica* infection and spread of amebiasis. The *Entamoeba* life cycle is essentially composed of the proliferating trophozoite and dormant cyst stages. Encystation and excystation are transition steps from trophozoites to cysts and vice versa. Transmission of amebiasis is solely mediated by cysts, and thus blocking encystation halts the spread of this infectious disease.

*E*. *histolytica* infection usually occurs by oral ingestion of cysts, the dormant form of the parasite (Fig 1) [5]. The cysts, particularly in endemic regions, can be found in various materials or on surfaces (including human hands) that have been contaminated with feces. This can directly or indirectly lead to oral intake. Because the cysts are resistant to severe environments (e.g., temperature, osmotic pressure, pH, and nutrient deprivation), they can pass through the stringent acidic conditions of the stomach and duodenum and reach the small intestine, where they hatch and become proliferative trophozoites. They then passively move to and colonize the large intestine and proliferate there. The proliferating trophozoites sometimes undergo progression to invasive steps that are closely associated with the clinical manifestations and pathogenesis of severe amebiasis [6,7]. Meanwhile, some proliferating trophozoites differentiate into cysts. The newly formed cysts, together with trophozoites, are then excreted during bowel movements. Only the excreted cysts are a source of infection because the trophozoites are labile to environmental assault. Hence, cysts are solely responsible for transmitting amebiasis, and blocking cyst formation halts the spread of this infectious disease to other individuals.

In this review, we focus on the transition step in the differentiation from the proliferative trophozoite into the dormant cyst: “encystation.” The life cycle of *E*. *histolytica* is essentially composed of the trophozoite and cyst stages, which are connected by encystation and excystation (see Fig 1). Encystation and excystation are fundamental cell differentiation processes and are, therefore, important from a biological as well as a medical perspective. Understanding the underlying molecular and cellular mechanisms will not only provide new insights into cellular differentiation but will also provide rationales and potential targets for the development of new preventive measures against amebiasis, such as drugs for blocking transmission (see Fig 1) [8,9]. Here, we take a medical perspective and discuss studies of *Entamoeba* encystation using inhibitory chemicals, and we categorize these chemicals based on the molecules they inhibit. We also present a perspective for developing transmission-blocking and prophylactic strategies against amebiasis.

## Molecules and Processes Involved in *Entamoeba* Encystation

All the studies described in this review have investigated *E*. *invadens*, a reptilian parasite, and not *E*. *histolytica*. This is because the in vitro culture of *E*. *invadens* has been adopted as a model system for encystation studies (Box 1) [8,10,11].

**Box 1. The In Vitro Culture of *E*. *invadens*: A Model System for the Study of Encystation**

**The in vitro culture of *E*. *invadens*:** The strains of *E*. *histolytica* available in the laboratory do not encyst after adaptation to culture conditions; however, *E*. *invadens* strains are able to undergo in vitro encystation. The *E*. *invadens* life cycle is the same as that of *E*. *histolytica*, and the symptoms caused by *E*. *invadens* infection are similar to those of *E*. *histolytica* [11,59].

In this system, encystation is induced by exposing *E*. *invadens* trophozoites to environmental changes that trigger encystation: carbon source deprivation [60], hypoosmotic shock [61], or both [11,62]. This usually involves the routine maintenance of trophozoites in Bisate-Iron-Serum-33 (BI-S-33) medium (a standard culture medium [26,63,64]) and transfer to encystation medium [11,22,52], which provides a decreased carbon source and a reduced osmotic level.

This model system, together with ever-expanding databases such as AmoebaDB, The National Center for Biotechnology Information (NCBI), and Kyoto Encyclopedia of Genes and Genomes (KEGG), has boosted our understanding of encystation.

## Gal-Terminated Ligands and Their Receptors

Distinct lines of evidence demonstrate that galactose (Gal)-terminated ligands and their receptors are involved in encystation:

1. Excess Gal, but not N-acetyl-galactosamine (GalNAc), impaired cyst formation when added in the in vitro encystation system [12,13]. Furthermore, excess Gal treatment made *E*. *invadens* form an aberrant cyst, termed a “wall-less cyst” [14].
2. Coculture of *E*. *invadens* trophozoites with the flagellate *Crithidia fasciculata* induced encystation. This induction was abolished by the addition of Gal to the culture medium or pretreatment of *Crithidia* with β-galactosidase, indicating the importance of the interaction between *Crithidia* and *Entamoeba* cells via Gal residues on the *Crithidia* surface [12].
3. Type III mucin or asialofetuin, both of which have a Gal residue as a terminal sugar, can substitute for adult bovine serum (ABS)—a critical component in the in vitro encystation system [9]—when used within an optimal concentration range (20 and 0.1–10 μg/mL, respectively). Their action is abolished by β-galactosidase pretreatment [13].

**Fig 2:**
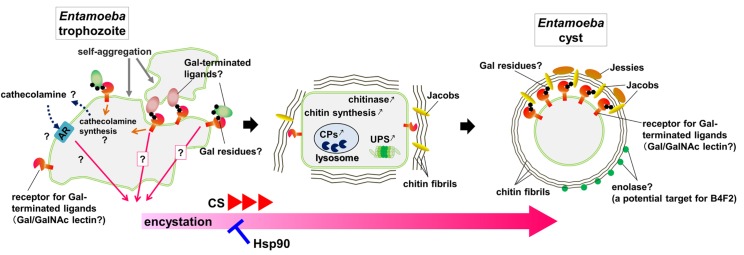
Schematic of proposed *Entamoeba* cyst formation. Depiction of the processes involved in *Entamoeba* cyst formation and their potential causal links. *Entamoeba* trophozoite self-aggregation via cell–cell interaction is an essential prerequisite process for encystation and is mediated by the binding of receptors to Gal-terminated ligands (left). Diverse pathways are implicated in the regulation of encystation (left and center). Numerous components are involved in forming intact cysts (right); some may be exclusive components in the cyst wall, and others may play versatile roles throughout the processes of cyst formation. Abbreviations used: AR, adrenergic receptor; B4F2, a monoclonal antibody; CP, cysteine protease; CS, cholesteryl sulfate; Gal, galactose; GalNAc, N-acetyl-galactosamine; Hsp90, heat shock protein 90; and UPS, ubiquitin proteasome system.

These findings indicate that Gal-terminated ligands are important for *Entamoeba* cell aggregation, a prerequisite for encystation [12,15,16]. They also suggest the presence of a cell surface molecule that mediates cell–cell interaction via binding to Gal-terminated ligands. Both the Gal-terminated ligands and their receptors await identification. However, *Entamoeba* Gal/GalNAc lectin is a candidate for the receptor of Gal-terminated ligands because it is among the best-characterized cell surface proteins of *Entamoeba* [1,17], it plays important roles in various processes of *E*. *histolytica* pathogenicity—including adherence, cytolysis, invasion, and resistance to lysis by complement—and it mediates binding to bacteria, red blood cells, epithelial cells, or mucin-coated tissues [16]. Hence, it is plausible that *Entamoeba* Gal/GalNAc lectin also participates in encystation [16]. Nevertheless, the following important issue needs to be confirmed: how do *Entamoeba* Gal-terminated ligands and their receptors commit a cell to encystation? (see Fig 2).

The importance of interactions of Gal-terminated ligands and their receptors has also been demonstrated for the formation of the *Entamoeba* cyst wall structure. Biochemical analyses identified Jacob as the most abundant glycoprotein in the cyst wall. Jacob can bind to the cyst wall component, chitin (see the “Chitin metabolism” section), and also to purified *E*. *histolytica* Gal/GalNAc lectin. Moreover, immunoelectron microscopy revealed that Jacob is released from multiple loci to the surface of encysting *E*. *invadens* cells, while low levels of Jacob are seen on the surface of “wall-less cysts” [14]. Another biochemical analysis showed that the protein Jessie can bind to chitin via its chitin-binding domain and also self-aggregate via its unique C-terminal domain [18]. Collectively, these data indicate that a cell surface Gal-lectin, Jacob, and Jessie are important protein components in the formation of an intact cyst (see Fig 2) [8,9,14,18]. However, several questions still remain to be answered (see Fig 2); for example, do Jacob and its family members indeed have a Gal residue as their terminal sugar? Are there any Gal-terminated ligands other than the Jacob family? Is *Entamoeba* Gal/GalNAc lectin indeed a receptor for Jacob and/or other putative Gal-terminated ligands that is needed to form the cyst wall structure?

Ultimately, elucidation of the molecular and cellular mechanisms to which Gal-terminated ligands and Gal-lectin are committed will provide potential targets and help develop strategies to control encystation and also inform new mechanistic insight in encystation.

## Autocrine Catecholamine System

Catecholamine is a vertebrate hormone synthesized from tyrosine and exerts crucial roles via adrenergic receptor (AR)-mediated signal transduction [19,20]. Coppi and colleagues demonstrated several lines of evidence that an autocrine catecholamine system is involved in *Entamoeba* encystation [21].

Catecholamines can be a substitute for ABS, a critical component in the in vitro encystation system [11], when used within an optimum concentration range; omitting ABS from the standard encystation medium decreased encystation efficiency to ~30%, while the addition of catecholamines to serum-free medium dose-dependently restored the efficiency to over 90%, which can be routinely obtained in the in vitro encystation system. A biphasic effect could be observed for epinephrine and norepinephrine; for example, epinephrine showed maximal activity at 1–10 μM and 1–10 mM.

Furthermore, both β-AR and β1-AR agonists could dose-dependently restore encystation efficiency when added to serum-free medium with a maximal level at 0.1 and 1 μM, respectively. In contrast, an α-AR agonist showed no significant effect in a concentration range of 0.01–10 μM. Consistently, only β-AR and β1-AR antagonists, but not α-AR and β2-AR antagonists, showed negative effects on encystation efficiency when added to standard encystation medium or to serum-free medium supplemented with epinephrine, a catecholamine. These results suggest the participation of the β1-AR–mediated catecholamine signaling pathway in encystation.

Results obtained by quantitative and qualitative biochemical analyses also suggest that *Entamoeba* trophozoite cells express an AR-like molecule on the cell surface and that they can produce catecholamines that are released into the medium during encystation [21]. Together, these results indicate that the *Entamoeba* autocrine catecholamine system uses β1-type receptors for encystation.

Nevertheless, the intriguing finding that autocrine regulation of catecholamine release via AR-mediated signal transduction plays a role in the cellular differentiation of a unicellular parasitic organism, *Entamoeba*, was inconsistent in a number of ways with the reported genome sequence (AmoebaDB). No counterparts of vertebrate ARs are encoded in the *Entamoeba* genome. Neither are any counterparts of vertebrate enzymes involved in the catecholamine synthetic pathway. These inconsistencies raise two critical questions; why do AR ligands, agonists, and antagonists all show significant, consistent (positive or negative) effects on encystation? How does *Entamoeba* produce catecholamines?

## Cholesteryl Sulfate Synthesis in Sulfur Metabolism

Cholesteryl sulfate (CS) is a common sulfate metabolite in mammals. Recently, CS was shown to play an important role in *Entamoeba* encystation [22].

Sulfur metabolism provides a variety of sulfur-containing biomolecules, such as methionine, cysteine, Fe-S clusters, sulfolipids, and sulfoproteins [23–25]. In *Entamoeba*, six sulfolipids (including CS) are synthesized as the terminal products of this metabolism [22]. Furthermore, CS enhanced encystation efficiency in the in vitro culture system within an optimum concentration range (50–200 μM). Conversely, inhibition of CS synthesis by chlorate—an inhibitor targeting ATP sulfurylase in sulfolipid synthesis—dose-dependently reduced encystation efficiency at >100 mM; this concentration range is sublethal for *E*. *invadens* trophozoite growth (IC_50_, 69.3 ± 6.6 mM). These lines of evidence imply that CS participates in the regulation of encystation and that factors involved in CS synthesis are potential targets for developing means to control encystation.

It is worth mentioning that the idea of CS playing an important role in the regulation of encystation provides an answer to the long-standing enigma of the role of *Entamoeba* mitosomes [22]. *Entamoeba* mitosomes are derived from canonical mitochondria and have largely lost typical mitochondrial functions during the course of eukaryote evolution; thus, their *raison d'être* and function have been a long-standing enigma (see Box 2) [22,26–29]. Furthermore, this unique mitosome-linked feature contributes to the adaptation of *Entamoeba* to its parasitic lifestyle (Box 2) [22].

**Box 2. *Entamoeba* Mitosomes Contribute to Parasitic Lifestyle Adaptation**

**A role of mitosomes:** Recently, *Entamoeba* mitosomes were shown to atypically compartmentalize sulfate activation and to be necessary for CS synthesis [26]. The regulation of encystation via CS synthesis is, therefore, an important role of *Entamoeba* mitosomes [22].

Furthermore, interestingly, this feature is not conserved in *Mastigamoeba*, a nonparasitic close relative of *Entamoeba*; *Mastigamoeba* likewise compartmentalizes sulfate activation into mitochondrion-related organelles, which have diversified from canonical mitochondria, but cannot synthesize CS because the cholesteryl sulfotransferase gene is absent in the genome [22,65]. These findings suggest that a unique feature of *Entamoeba* mitosomes contributes to its adaptation to its parasitic lifestyle.

Hence, elucidation of the underlying molecular and cellular mechanisms modulated by CS will provide not only a new mechanistic insight into encystation but also a new paradigm for linking organellology and evolutionary adaptation to parasitism.

## Hsp90

In the process of encystation, negative as well as positive factors are involved. Heat shock protein 90 (Hsp90) is such a negative regulator of encystation [30]. Hsp90 is a chaperone that is highly conserved from bacteria to mammals and plays crucial roles via interaction with co-chaperones [31–34]. Protozoan parasites are, however, an exception; they maintain the conserved Hsp90 but lack some co-chaperones. Indeed, *E*. *histolytica* has a highly conserved Hsp90 but lacks the co-chaperones p23, cyclophilin 40 (Cyp40), cell division cycle 37 (Cdc37), and full-length Activator of the Hsp90 ATPase-1 (Aha1), although it does possess a novel truncated Aha1 [32,35]. Singh et al. demonstrated that the ATPase activity of purified recombinant *E*. *histolytica* Hsp90 (EhHsp90) was inhibited by 17-allylamino-17-demethoxygeldanamycin (17-AAG), a well-known inhibitor of Hsp90 with an IC_50_ value of 30.9 μM [35,36]. Furthermore, 17-AAG inhibited the growth of *E*. *histolytica* trophozoites with an IC_50_ value of 546 nM. These results suggest that 17-AAG targets native EhHsp90 in vivo and that EhHsp90 plays an indispensable role in *E*. *histolytica* trophozoites [35].

Hsp90 has recently been shown to play a role in encystation [30]. Encystation efficiency was enhanced by almost two-fold by pretreatment of *E*. *invadens* with 600 nM 17-AAG for 24 hours prior to inducing encystation; the concentration used is a sublethal dose for *E*. *invadens* trophozoite growth (IC_50_, 711 nM). Consistently, mRNA levels of Hsp90 and co-chaperones are dramatically down-regulated during encystation, suggesting that Hsp90 acts as a negative regulator of encystation. Hsp90, therefore, appears to play versatile roles in the parasitic life cycle of *Entamoeba*, and elucidation of the underlying molecular and cellular mechanisms in which Hsp90 and co-chaperones participate will inform numerous topics on parasitism as well as differentiation.

## Chitin Metabolism

Chitin metabolism may provide potential targets for developing strategies to block the transmission of amebiasis because chitin is present in the cyst wall [5,8,9,15,18] and is not a component of mammalian cells [37,38]. Two independent groups published inconsistent results; one study showed that polyoxin D and Nikkomycin (structural analogs of uridine diphosphate-N-acetylglucosamine, a substrate for chitin synthase) inhibited the cyst formation in a dose-dependent manner when added to the in vitro culture (2–500 and 10–50 μg/mL, respectively) [39]. The other study showed that chitin synthase activity in *E*. *invadens* cyst lysates was not inhibited by polyoxin D or Nikkomycin even at 100 μg/mL [40]. This inconsistency raises two questions: Is the *Entamoeba* chitin synthase a target of polyoxin D and Nikkomycin? Is the *Entamoeba* chitin synthase a redundant enzyme? The *Entamoeba* chitin synthase may indeed be redundant because two chitin synthases are encoded in the genome (AmoebaDB); however, the critical question of what is the target of polyoxin D and Nikkomycin remains unanswered.

The importance of the chitin anabolism during encystation is underscored by a study exploiting the *Entamoeba* database (AmoebaDB) and a gene knockdown approach [41]. All of the transcripts for the putative genes encoding the enzymes in this pathway are up-regulated during encystation. Moreover, the addition of a double-stranded RNA encoding part of an enzyme in this pathway, glucosamine-6-phosphate isomerase, to the in vitro culture reduces its mRNA level, resulting in significant impairment of chitin synthesis. Furthermore, the addition of glycogen phosphorylase inhibitor to the encystation-inducing in vitro culture reduced the chitin level of cysts; glycogen phosphorylase supplies glucose to chitin synthesis by the breakdown of glycogen. Nevertheless, a causal link between the level of chitin synthesis and encystation efficacy is not currently substantiated.

As well as chitin anabolism, the importance of its catabolism has been demonstrated [42]. The addition of allosamidin, an inhibitor of chitinase, to the culture delayed the progression of the early phase of encystation but could not abolish cyst formation in the in vitro culture. In addition, chitinase activity in *E*. *invadens* cell extracts prepared from the encystation-inducing in vitro culture was inhibited by allosamidin with a Ki value of 65 nM. The effect on the ability of cells to form cysts was observed within a concentration range of 80–320 μM, more than three orders of magnitude higher than its Ki value on the enzyme. It is, therefore, necessary to determine whether the target of allosamidin is chitinase. Ultimately, unraveling the involvement of chitin metabolism in encystation is needed.

## Proteolytic Systems: Lysosomes and Ubiquitin Proteasome System

In eukaryotes, two proteolytic systems—i.e., the lysosome system and the ubiquitin (Ub) proteasome system (UPS)—control intracellular protein levels within optimal ranges [43,44]. In *Entamoeba*, these two systems play important roles in encystation as well as in proliferation of trophozoites and pathogenicity [1,45].

### (1) Cysteine proteases in lysosomes

Involvement of lysosomes in encystation was shown by using specific irreversible cysteine protease (CP) inhibitors, Z-Phe-Arg-CH_2_F (benzyloxycarbonyl phenylalanyl arginyl fluoromethyl ketone) and E64; CP is known as an important virulence factor in *Entamoeba* [46–49]. Sharma et al. described the significant ability of Z-Phe-Arg-CH_2_F to reduce cyst formation efficiency in the in vitro encystation system [46].

Gonzalez et al. demonstrated that the addition of 20 μM E64 significantly delayed the progression of the early phase of encystation but could not abolish cyst formation in the in vitro culture [47,50]. This indicates that CPs only partially contribute to encystation. Alternatively, it implies that the E64 inhibitory effect was not fully exerted in the reported experimental conditions; during incubation, inadequately supplemented E64 was titrated by the turnover of E64-bound CPs and/or by the presence of different types of E64-sensitive proteolytic enzymes, resulting in abrogation of the inhibitory effect. In the studies using CP inhibitors, indirect effects on encystation caused by lysosome dysfunction in trophozoites also needs to be considered [51].

Among 64 CP genes encoded in the *E*. *invadens* genome (AmoebaDB), 11 CP mRNAs were up-regulated upon induction of encystation in vitro [52]. In the cysts isolated from amebiasis patients, 8 *E*. *histolytica* CP mRNAs were also up-regulated, 2 of which were counterparts of the CP genes detected in the above-mentioned 11 *E*. *invadens* genes [52,53]. Collectively, these results indicate that CP plays an important role in encystation, although the nature of this involvement requires elucidation.

### (2) Ubiquitin proteasome system

The UPS, another essential intracellular proteolytic system, is typically composed of sequential chain reactions, comprising Ub activation, conjugation, ligation, and elongation, as well as protein degradation in the proteasome, a protein complex containing various proteases [44,54]. This proteolytic system also has an important influence on encystation.

Eichinger and colleagues substantiated the involvement of the UPS in encystation [47]. Lactacystin, a specific proteasome inhibitor, blocked cyst formation in a dose-dependent manner with an IC_50_ value of 1.25–2.5 μM when added in the in vitro culture. Enrichment of the lactacystin-inhibitable, chymotrypsin-like activity was achieved by column chromatography of the lactacystin-treated *E*. *invadens* cell lysate. The enriched fractions show trypsin-like and peptidyl-glutamyl peptide-hydrolyzing (caspase-like) activities. All three of these activities were inhibited by lactacystin at ≤50 μM, which is characteristic of the proteasome. Furthermore, the fractions display typical features of eukaryotic 20S proteasome core subunits—i.e., molecular masses and isoelectric points of major UPS proteins and typical structures as visualized by negatively stained electron microscopy. These studies confirm that, similarly to other organisms, the proteasome in *Entamoeba* is the target of lactacystin and that the proteasome plays an important role in encystation.

The transcriptional up-regulation of the Ub gene occurred coincidently with other encystation-specific genes (e.g., gene 122 and chitinase 1) 24 hours after the initiation of encystation in vitro [47]. Moreover, β-lactone and MG-132, specific proteasome inhibitors, also dose-dependently inhibited cyst formation in the in vitro culture, with IC_50_ values of ~1.1 and ~60 μM, respectively [45]. These results further indicate that the UPS plays an important role in encystation. Nevertheless, it cannot be ruled out that overall trophozoite health is affected by proteasome dysfunction that indirectly causes decreased efficiency of cyst formation.

A gene previously identified to encode the *E*. *invadens* Ub (AF016643) [47] does not exist in AmoebaDB. Furthermore, a gene annotated as the Ub gene in the AmoebaDB (EIN_063840) does not show high amino acid sequence conservation with other Ubs from different organisms. Therefore, identifying bona fide *Entamoeba* Ub genes is necessary to address the important issue of how the UPS is involved in encystation.

## Enolase: A Potential Target for a Monoclonal Antibody (B4F2)

Segovia-Gamboa et al. reported the isolation of a monoclonal antibody, B4F2, by screening monoclonal antibodies raised against *E*. *invadens* intact cysts that could inhibit encystation in the in vitro system [55]. The movement of B4F2’s target molecule during encystation was also shown. Moreover, enolase was demonstrated to be a potential target for B4F2. These findings indicate a role of enolase, a glycolytic enzyme, in encystation; however, further study is needed to confirm whether the B4F2 antigen is indeed enolase.

## Conclusions and Future Perspectives

Amebiasis is a serious public health problem; therefore, the development of novel strategies to manipulate *E*. *histolytica* encystation is important for realizing new preventive measures. Impairing the ability to form cysts can block the spread of the disease because dormancy of the *E*. *histolytica* cyst is crucial for disease transmission. This type of approach cannot directly eliminate the causative agent, the trophozoite, from infected patients, so it would not cure amebiasis. However, it could be an effective approach to mitigate the disease because high numbers of asymptomatic patients who do not require clinical treatment are unconsciously spreading the disease (see Fig 1). A combination approach of eliminating the causative agent itself and interrupting its life cycle would be effective against infectious diseases such as amebiasis and would accelerate a reduction in endemicity.

Most of the molecules described and discussed in this review are potential targets for the development of transmission-blocking strategies. Ideal targets are molecules that exist exclusively in the parasite and not in the host. However, enzymes that have human and *E*. *histolytica* counterparts can also be considered as targets if they have distinct features. These features include differences of primary structure, which can be useful for candidate selection (Table 1), and enzyme characteristics, such as substrate specificity, catalytic mechanism, and three-dimensional structures, which are relevant to drug design. Another important factor is the organs and tissues in which the human enzymes are expressed because this has a large bearing on the most efficient drug delivery system. Thus, it will be necessary to differentiate various characteristics of *E*. *histolytica* enzymes from their human counterparts.

**Table 1:** Relevant information on proteins described in this review.

Described in	Protein	Specific inhibitor	*Entamoeba histolytica*	Human counterpart	Percentage amino acid sequence identity
**Gal-terminated ligands and their receptors**	**Gal/GalNAc lectin**		**+**	**-**	
**Autocrine catecholamine system**	**catecholamine synthetic pathway**		**-?**	**+**	
	**adrenergic receptor**		**-?**	**+**	
**Cholesteryl sulfate synthesis in sulfur metabolism**	**cholesteryl sulfotransferase**		**XP_649714**	**NP_814444**	**no homology**
	**ATP sulfurylase**	**chlorate**	**XP_653570**	**NP_005434**	**26.5%**
**Hsp90**	**Hsp90**	**17-AAG**	**XP_653132**	**NP_005339**	**64.1%**
**Chitin metabolism**	**chitin synthesis pathway**		**+**	**-**	
	**chitinase**	**allosamidin**	**XP_652205**	**AAI05681**	**39.7%**
**Proteolytic systems**	**proteasome subunit beta type 5**	**lactacystin**	**XP_653800**	**NP_002788**	**44.8%**
	**ubiquitin**		**XP_650163**	**EAX04503**	**89.5%**
**A potential target for a monoclonal antibody (B4F2)**	**enolase?**		**XP_649161**	**NP_001966**	**62.4%**

The studies reviewed also describe the important molecular processes involved in encystation; however, the underlying mechanisms are not completely understood. The processes described appear to function separately, and it is necessary to determine causal connections to produce an orchestrated network for the regulation of cyst formation (Fig 2). As described in this review, chemicals that can target specific molecules participating in cyst formation can be effective; nevertheless, a major concern is whether their effects result directly from inhibition of target molecules that specifically function in encystation or indirectly because the target molecule also plays important roles in trophozoite proliferation. For example, some target molecules may be involved in fundamental processes of cell maintenance throughout the *Entamoeba* life cycle (see the “Proteolytic systems” section; [56–58]). This concern can be addressed using a combination approach of gene knockdown (gene knockout has not been achieved in *Entamoeba*) and supplementation with the metabolite produced by a target enzyme.

Further molecular characterization and elucidation of the underlying mechanism of encystation, both in vitro and in vivo, are essential to provide a solid scientific basis to enable translation of research knowledge into clinically useful materials (see Fig 2). As the ultimate goal, prophylactic drugs as well as transmission-blocking drugs and vaccines against amebiasis will hopefully be developed in the future.

## References

[1] Ralston KS, Petri WA. The ways of a killer: how does *Entamoeba histolytica* elicit host cell death? Essays Biochem. 2011;51:193–210. 10.1042/bse0510193 22023450

[2] Haque R, Huston CD, Hughes M, Houpt E, Petri WA Jr. Amebiasis. N Engl J Med. 2003;348(16):1565–73. . 12700377 10.1056/NEJMra022710

[3] Quach J, St-Pierre J, Chadee K. The future for vaccine development against *Entamoeba histolytica* . Hum Vaccin Immunother. 2014;10(6):1514–21. 10.4161/hv.27796 24504133 PMC5396225

[4] Watanabe K, Petri WA Jr. Molecular biology research to benefit patients with *Entamoeba histolytica* infection. Mol Microbiol. 2015;98(2):208–17. 10.1111/mmi.13131 26173474

[5] Samuelson J, Bushkin GG, Chatterjee A, Robbins PW. Strategies to discover the structural components of cyst and oocyst walls. Eukaryot Cell. 2013;12(12):1578–87. 10.1128/EC.00213-13 24096907 PMC3889564

[6] Moonah SN, Jiang NM, Petri WA Jr. Host immune response to intestinal amebiasis. PLoS Pathog. 2013;9(8):e1003489 10.1371/journal.ppat.1003489 23990778 PMC3749964

[7] Begum S, Quach J, Chadee K. Immune Evasion Mechanisms of *Entamoeba histolytica*: Progression to Disease. Front Microbiol. 2015;6:1394 10.3389/fmicb.2015.01394 26696997 PMC4678226

[8] Aguilar-Diaz H, Carrero JC, Arguello-Garcia R, Laclette JP, Morales-Montor J. Cyst and encystment in protozoan parasites: optimal targets for new life-cycle interrupting strategies? Trends Parasitol. 2011;27(10):450–8. 10.1016/j.pt.2011.06.003 21775209

[9] Samuelson J, Robbins P. A simple fibril and lectin model for cyst walls of *Entamoeba* and perhaps *Giardia* . Trends Parasitol. 2011;27(1):17–22. 10.1016/j.pt.2010.09.002 20934911 PMC3014499

[10] Eichinger D. Encystation in parasitic protozoa. Curr Opin Microbiol. 2001;4(4):421–6. 10.1016/s1369-5274(00)00229-0 . 11495805

[11] Sanchez L, Enea V, Eichinger D. Identification of a developmentally regulated transcript expressed during encystation of *Entamoeba invadens* . Mol Biochem Parasitol. 1994;67(1):125–35. 10.1016/0166-6851(94)90102-3 . 7838173

[12] Cho J, Eichinger D. Crithidia fasciculata induces encystation of *Entamoeba invadens* in a galactose-dependent manner. J Parasitol. 1998;84(4):705–10. 10.2307/3284574 . 9714198

[13] Coppi A, Eichinger D. Regulation of *Entamoeba invadens* encystation and gene expression with galactose and N-acetylglucosamine. Mol Biochem Parasitol. 1999;102(1):67–77. 10.1016/s0166-6851(99)00085-7 . 10477177

[14] Frisardi M, Ghosh SK, Field J, Van Dellen K, Rogers R, Robbins P, et al The most abundant glycoprotein of amebic cyst walls (Jacob) is a lectin with five Cys-rich, chitin-binding domains. Infect Immun. 2000;68(7):4217–24. 10.1128/iai.68.7.4217-4224.2000 . 10858239 PMC101730

[15] Eichinger D. Encystation of entamoeba parasites. Bioessays. 1997;19(7):633–9. 10.1002/bies.950190714 . 9230696

[16] Eichinger D. A role for a galactose lectin and its ligands during encystment of *Entamoeba* . J Eukaryot Microbiol. 2001;48(1):17–21. 10.1111/j.1550-7408.2001.tb00411.x . 11249188

[17] Aguirre Garcia M, Gutierrez-Kobeh L, Lopez Vancell R. *Entamoeba histolytica*: adhesins and lectins in the trophozoite surface. Molecules. 2015;20(2):2802–15. 10.3390/molecules20022802 25671365 PMC6272351

[18] Chatterjee A, Ghosh SK, Jang K, Bullitt E, Moore L, Robbins PW, et al Evidence for a "wattle and daub" model of the cyst wall of entamoeba. PLoS Pathog. 2009;5(7):e1000498 10.1371/journal.ppat.1000498 19578434 PMC2698119

[19] Caron MG, Lefkowitz RJ. Catecholamine receptors: structure, function, and regulation. Recent Prog Horm Res. 1993;48:277–90. . 8441851 10.1016/b978-0-12-571148-7.50014-2

[20] Docherty JR. Subtypes of functional alpha1- and alpha2-adrenoceptors. Eur J Pharmacol. 1998;361(1):1–15. . 9851536 10.1016/s0014-2999(98)00682-7

[21] Coppi A, Merali S, Eichinger D. The enteric parasite *Entamoeba* uses an autocrine catecholamine system during differentiation into the infectious cyst stage. J Biol Chem. 2002;277(10):8083–90. 10.1074/jbc.M111895200 . 11779874

[22] Mi-ichi F, Miyamoto T, Takao S, Jeelani G, Hashimoto T, Hara H, et al *Entamoeba* mitosomes play an important role in encystation by association with cholesteryl sulfate synthesis. Proc Natl Acad Sci U S A. 2015;112(22):E2884–90. 10.1073/pnas.1423718112 25986376 PMC4460517

[23] Yoshinari K, Petrotchenko EV, Pedersen LC, Negishi M. Crystal structure-based studies of cytosolic sulfotransferase. J Biochem Mol Toxicol. 2001;15(2):67–75. . 11284047 10.1002/jbt.1

[24] Rath VL, Verdugo D, Hemmerich S. Sulfotransferase structural biology and inhibitor discovery. Drug Discov Today. 2004;9(23):1003–11. . 15574316 10.1016/S1359-6446(04)03273-8

[25] Koprivova A, Kopriva S. Molecular mechanisms of regulation of sulfate assimilation: first steps on a long road. Front Plant Sci. 2014;5:589 10.3389/fpls.2014.00589 25400653 PMC4212615

[26] Mi-ichi F, Abu Yousuf M, Nakada-Tsukui K, Nozaki T. Mitosomes in *Entamoeba histolytica* contain a sulfate activation pathway. Proc Natl Acad Sci U S A. 2009;106(51):21731–6. 10.1073/pnas.0907106106 19995967 PMC2799805

[27] Mi-ichi F, Nozawa A, Yoshida H, Tozawa Y, Nozaki T. Evidence that the *Entamoeba histolytica* Mitochondrial Carrier Family Links Mitosomal and Cytosolic Pathways through Exchange of 3'-Phosphoadenosine 5'-Phosphosulfate and ATP. Eukaryot Cell. 2015;14(11):1144–50. 10.1128/EC.00130-15 26385892 PMC4621310

[28] Aguilera P, Barry T, Tovar J. *Entamoeba histolytica* mitosomes: organelles in search of a function. Exp Parasitol. 2008;118(1):10–6. . 17880942 10.1016/j.exppara.2007.08.004

[29] van der Giezen M. Hydrogenosomes and mitosomes: conservation and evolution of functions. J Eukaryot Microbiol. 2009;56(3):221–31. 10.1111/j.1550-7408.2009.00407.x 19527349

[30] Singh M, Sharma S, Bhattacharya A, Tatu U. Heat Shock Protein 90 regulates encystation in *Entamoeba* . Front Microbiol. 2015;6:1125 10.3389/fmicb.2015.01125 26528271 PMC4602144

[31] Sidera K, Patsavoudi E. HSP90 inhibitors: current development and potential in cancer therapy. Recent Pat Anticancer Drug Discov. 2014;9(1):1–20. . 23312026

[32] Singh C, Atri N. Chemo-informatic design of antibiotic geldenamycin analogs to target stress proteins HSP90 of pathogenic protozoan parasites. Bioinformation. 2013;9(7):329–33. 10.6026/97320630009329 23750075 PMC3669783

[33] Didenko T, Duarte AM, Karagoz GE, Rudiger SG. Hsp90 structure and function studied by NMR spectroscopy. Biochim Biophys Acta. 2012;1823(3):636–47. 10.1016/j.bbamcr.2011.11.009 22155720

[34] Johnson JL, Brown C. Plasticity of the Hsp90 chaperone machine in divergent eukaryotic organisms. Cell Stress Chaperones. 2009;14(1):83–94. 10.1007/s12192-008-0058-9 18636345 PMC2673905

[35] Singh M, Shah V, Tatu U. A novel C-terminal homologue of Aha1 co-chaperone binds to heat shock protein 90 and stimulates its ATPase activity in *Entamoeba histolytica* . J Mol Biol. 2014;426(8):1786–98. 10.1016/j.jmb.2014.01.008 24486610

[36] Isaacs JS, Xu W, Neckers L. Heat shock protein 90 as a molecular target for cancer therapeutics. Cancer Cell. 2003;3(3):213–7. . 12676580 10.1016/s1535-6108(03)00029-1

[37] Lee CG, Da Silva CA, Dela Cruz CS, Ahangari F, Ma B, Kang MJ, et al Role of chitin and chitinase/chitinase-like proteins in inflammation, tissue remodeling, and injury. Annu Rev Physiol. 2011;73:479–501. 10.1146/annurev-physiol-012110-142250 21054166 PMC3864643

[38] Kanneganti M, Kamba A, Mizoguchi E. Role of chitotriosidase (chitinase 1) under normal and disease conditions. J Epithel Biol Pharmacol. 2012;5:1–9. . 23439988 10.2174/1875044301205010001 PMC3579558

[39] Avron B, Deutsch RM, Mirelman D. Chitin synthesis inhibitors prevent cyst formation by *Entamoeba* trophozoites. Biochem Biophys Res Commun. 1982;108(2):815–21. . 7150326 10.1016/0006-291x(82)90902-0

[40] Das S, Gillin FD. Chitin synthase in encysting *Entamoeba invadens* . Biochem J. 1991;280 (Pt 3):641–7. . 1764027 10.1042/bj2800641 PMC1130502

[41] Samanta SK, Ghosh SK. The chitin biosynthesis pathway in *Entamoeba* and the role of glucosamine-6-P isomerase by RNA interference. Mol Biochem Parasitol. 2012;186(1):60–8. 10.1016/j.molbiopara.2012.09.011 23058929

[42] Villagomez-Castro JC, Calvo-Mendez C, Lopez-Romero E. Chitinase activity in encysting *Entamoeba invadens* and its inhibition by allosamidin. Mol Biochem Parasitol. 1992;52(1):53–62. . 1625707 10.1016/0166-6851(92)90035-i

[43] Korolchuk VI, Menzies FM, Rubinsztein DC. Mechanisms of cross-talk between the ubiquitin-proteasome and autophagy-lysosome systems. FEBS Lett. 2010;584(7):1393–8. 10.1016/j.febslet.2009.12.047 20040365

[44] Pickart CM. Back to the future with ubiquitin. Cell. 2004;116(2):181–90. . 14744430 10.1016/s0092-8674(03)01074-2

[45] Makioka A, Kumagai M, Ohtomo H, Kobayashi S, Takeuchi T. Effect of proteasome inhibitors on the growth, encystation, and excystation of *Entamoeba histolytica* and *Entamoeba invadens* . Parasitol Res. 2002;88(5):454–9. . 12049464 10.1007/s00436-002-0601-z

[46] Sharma M, Hirata K, Herdman S, Reed S. *Entamoeba invadens*: characterization of cysteine proteinases. Exp Parasitol. 1996;84(1):84–91. . 8888735 10.1006/expr.1996.0092

[47] Gonzalez J, Bai G, Frevert U, Corey EJ, Eichinger D. Proteasome-dependent cyst formation and stage-specific ubiquitin mRNA accumulation in *Entamoeba invadens* . Eur J Biochem. 1999;264(3):897–904. . 10491138 10.1046/j.1432-1327.1999.00682.x

[48] Garcia-Calvo M, Peterson EP, Leiting B, Ruel R, Nicholson DW, Thornberry NA. Inhibition of human caspases by peptide-based and macromolecular inhibitors. J Biol Chem. 1998;273(49):32608–13. . 9829999 10.1074/jbc.273.49.32608

[49] Que X, Reed SL. Cysteine proteinases and the pathogenesis of amebiasis. Clin Microbiol Rev. 2000;13(2):196–206. . 10755997 10.1128/cmr.13.2.196-206.2000 PMC100150

[50] Gonzalez J, Frevert U, Corey EJ, Nussenzweig V, Eichinger D. Proteasome function is required for encystation of *Entamoeba invadens* . Arch Med Res. 1997;28 Spec No:139–40. . 9033045

[51] de Meester F, Shaw E, Scholze H, Stolarsky T, Mirelman D. Specific labeling of cysteine proteinases in pathogenic and nonpathogenic *Entamoeba histolytica* . Infect Immun. 1990;58(5):1396–401. . 2323821 10.1128/iai.58.5.1396-1401.1990 PMC258638

[52] De Cadiz AE, Jeelani G, Nakada-Tsukui K, Caler E, Nozaki T. Transcriptome analysis of encystation in *Entamoeba invadens* . PLoS ONE. 2013;8(9):e74840 10.1371/journal.pone.0074840 24040350 PMC3770568

[53] Ehrenkaufer GM, Haque R, Hackney JA, Eichinger DJ, Singh U. Identification of developmentally regulated genes in *Entamoeba histolytica*: insights into mechanisms of stage conversion in a protozoan parasite. Cell Microbiol. 2007;9(6):1426–44. 10.1111/j.1462-5822.2006.00882.x . 17250591

[54] Baumeister W, Walz J, Zuhl F, Seemuller E. The proteasome: paradigm of a self-compartmentalizing protease. Cell. 1998;92(3):367–80. . 9476896 10.1016/s0092-8674(00)80929-0

[55] Segovia-Gamboa NC, Chavez-Munguia B, Medina-Flores Y, Cazares-Raga FE, Hernandez-Ramirez VI, Martinez-Palomo A, et al *Entamoeba invadens*, encystation process and enolase. Exp Parasitol. 2010;125(2):63–9. 10.1016/j.exppara.2009.12.019 20045689

[56] Makioka A, Kumagai M, Kobayashi S, Takeuchi T. Involvement of signaling through protein kinase C and phosphatidylinositol 3-kinase in the excystation and metacystic development of *Entamoeba invadens* . Parasitol Res. 2003;91(3):204–8. 10.1007/s00436-003-0955-x . 12923632

[57] Makioka A, Kumagai M, Kobayashi S, Takeuchi T. Different effects of cytochalasins on the growth and differentiation of *Entamoeba invadens* . Parasitol Res. 2004;93(1):68–71. 10.1007/s00436-004-1106-8 . 15103555

[58] Ehrenkaufer GM, Weedall GD, Williams D, Lorenzi HA, Caler E, Hall N, et al The genome and transcriptome of the enteric parasite *Entamoeba invadens*, a model for encystation. Genome Biol. 2013;14(7):R77 10.1186/gb-2013-14-7-r77 23889909 PMC4053983

[59] Kojimoto A, Uchida K, Horii Y, Okumura S, Yamaguch R, Tateyama S. Amebiasis in four ball pythons, *Python reginus* . J Vet Med Sci. 2001;63(12):1365–8. . 11789622 10.1292/jvms.63.1365

[60] Vazquezdelara-Cisneros LG, Arroyo-Begovich A. Induction of encystation of *Entamoeba invadens* by removal of glucose from the culture medium. J Parasitol. 1984;70(5):629–33. . 6512629

[61] Bailey GB, Rengypian S. Osmotic stress as a factor controlling encystation of *Entamoeba invadens* . Arch Invest Med (Mex). 1980;11(1 Suppl):11–6. . 7469635

[62] Avron B, Stolarsky T, Chayen A, Mirelman D. Encystation of *Entamoeba invadens* IP-1 is induced by lowering the osmotic pressure and depletion of nutrients from the medium. J Protozool. 1986;33(4):522–5. . 3795143 10.1111/j.1550-7408.1986.tb05655.x

[63] Diamond LS, Harlow DR, Cunnick CC. A new medium for the axenic cultivation of *Entamoeba histolytica* and other *Entamoeba* . Trans R Soc Trop Med Hyg. 1978;72(4):431–2. . 212851 10.1016/0035-9203(78)90144-x

[64] Deloer S, Nakamura R, Mi-ichi F, Adachi K, Kobayashi S, Hamano S. Mouse models of amoebiasis and culture methods of amoeba. Parasitol Int. 2016;65(5):520–5. 10.1016/j.parint.2016.03.012 27080249

[65] Nyvltova E, Stairs CW, Hrdy I, Ridl J, Mach J, Paces J, et al Lateral gene transfer and gene duplication played a key role in the evolution of *Mastigamoeba balamuthi* hydrogenosomes. Mol Biol Evol. 2015;32(4):1039–55. 10.1093/molbev/msu408 25573905 PMC4379409

